# Valvular Heart Diseases in Swedish Males and Females: A National Cohort Study

**DOI:** 10.1016/j.jacadv.2026.102666

**Published:** 2026-03-19

**Authors:** Per Wändell, Xinjun Li, Axel C. Carlsson, Jan Sundquist, Kristina Sundquist

**Affiliations:** a Division of Family Medicine and Primary Care, Department of Neurobiology, Care Sciences and Society, Karolinska Institutet, Huddinge, Sweden; b Center for Primary Health Care Research, Department of Clinical Sciences Malmö, Lund University, Malmö, Sweden; c Academic Primary Health Care Centre, Region Stockholm, Sweden; d University Clinic Primary Care, Skåne University Hospital, Malmö, Sweden; e Department of Family and Community Medicine, McGovern Medical School, The University of Texas Health Science Center, Houston, Texas, USA

**Keywords:** immigrants, neighborhood, rheumatic valvular heart disease, sex, socioeconomic status, valvular heart disease

## Abstract

**Background:**

Sex differences in valvular heart diseases have been examined but rarely using a nationwide, comprehensive approach.

**Objectives:**

The aim of this study was to analyze the risk of different types of valvular heart diseases among males and females in Sweden.

**Methods:**

This was a nationwide Swedish study of all individuals (N = 9,984,758; 4,969,472 males and 5,015,286 females). Valvular heart diseases were defined as at least 1 registered diagnosis in the National Patient Register between January 1, 1998, and December 31, 2018. Cox regression analysis was used to estimate HRs with 95% CIs of incident valvular heart diseases in males vs females. The Cox regression models were adjusted for age, comorbidities, and sociodemographic factors.

**Results:**

There were 111,315 male cases and 107,527 female cases, corresponding to overall incidence rates per 100,000 person-years of 68.6 (95% CI: 67.5-69.8) among males and 51.3 (95% CI: 50.6-51.9) among females. The HRs (with 95% CI) for males compared to females were for rheumatic mitral valve disorders 0.50 (0.46-0.53), for rheumatic aortic valve disorders 1.08 (0.99-1.18), for rheumatic tricuspid valve disorders 0.61 (0.57-0.66), for nonrheumatic mitral valve disorders 1.08 (1.06-1.10), for nonrheumatic aortic valve disorders 1.25 (1.24-1.27), for nonrheumatic tricuspid valve disorders 0.72 (0.68-0.77), and for pulmonary valve disorders 0.82 (0.75-0.90).

**Conclusions:**

We observed certain sex differences, with, in general, a higher incidence rate among males of valvular heart disease diagnoses, but a lower risk of specific valve disorders. The underlying factors for the sex differences are unclear and need further investigation.

Valvular heart diseases are important causes of congestive heart failure. In addition, they are also among the heart disorders that are possible to operate on surgically and therefore important to diagnose. Worldwide, valvular heart diseases have the highest rates in Western high-income countries and Asian Pacific high-income countries, and lower rates in other regions of the world, albeit varying in East Asia.^1^ In high-income countries, functional and degenerative diseases are predominant, while in low- and middle-income countries, there is a predominance of rheumatic heart diseases.^2^

Valvular heart diseases were historically mainly caused by rheumatic heart diseases as a consequence of infections, often by streptococci. However, acute rheumatic fever has decreased during the last decades in industrialized or developed countries, while still being present in less developed parts of the world.^3^ A study from Soweto, South Africa, showed that “historically” prevalent heart diseases, with primary valvular heart diseases predominating within this group (62%), were more common in individuals younger than 50 years of age, females, and individuals belonging to the majority population.^4^

The differences between males and females are of special interest. A global study showed a higher risk among females for rheumatic heart disease and degenerative mitral heart disease, but a slight male predominance for calcific aortic valve disease.^5^ A Canadian review found that among males, a higher risk of bicuspid aortic valve disorders was present, but among females, degenerative aortic valve disease and mitral and tricuspid valve diseases were more common.^6^ An Indian study of rheumatic heart diseases found a female predominance for mitral and tricuspid valve diseases, but a male predominance for aortic valve diseases.^7^ A European study of severe congenital valvular heart diseases found a male predominance overall, as well as for degenerative and congenital valvular diseases, secondary mitral regurgitation, aortic valve diseases, and mitral regurgitation, but a female predominance for mitral valve diseases.^8^ A Danish study found a higher risk among males for aortic valve disorders, and also a slightly higher risk for mitral regurgitation.^9^ A Swedish study on valvular heart diseases among immigrants found a male predominance overall and for nonrheumatic mitral and aortic valve disorders, but a female predominance for rheumatic mitral valve diseases and tricuspid valve diseases, both rheumatic and nonrheumatic, and also for pulmonary valve disorders.^10^ Another Swedish study showed higher rates among males for aortic valve disorders, mitral regurgitation, and pulmonary regurgitation.^11^

Regarding age, a Fijian study found a female predominance of rheumatic heart diseases,^12^ while a study from Nicaragua found no difference among children but a higher risk for females among young adults.^13^ A Japanese study of severe aortic stenosis found a female predominance that was increasing with age, but more males in the ages ≤64 years had severe aortic stenosis.^14^ The existing literature is spurious, and we believe that Swedish nationwide registers covering the total population are a good opportunity to unravel potential sex differences in different types of valvular heart diagnoses.

The aim of this study was to estimate the risk of valvular heart diseases in general among males and females in Sweden, taking several covariates into account. We also aimed to study sex differences in rheumatic and nonrheumatic valvular heart diseases separately.

## Methods

We used national Swedish registers, that is, the National Patient Register (NPR) and the Swedish Total Population Register. The NPR includes diagnoses from all Swedish hospitals, that is, for in-hospital patients since 1987 and for outpatients from 2001 onward. The Total Population Register includes data on country of origin and sociodemographic factors on all residents of Sweden with a residence permit. The study period started on January 1, 1998, which represented baseline, and ended on December 31, 2018. The outcomes were defined as the first diagnosis recorded within the study period, either inpatient or outpatient encounters.

### Study population

All individuals in Sweden were included (N = 9,984,758; 4,969,472 males and 5,015,286 females), and also subdivided by age groups (0-49, 50-59, 60-69, 70-79, and ≥80 years) as regard age-specific incidence rates for all valvular heart diseases. Individuals with a diagnosis of a valvular heart disease diagnosed before 1997 were excluded, as we restricted the results to International Classification of Diseases (ICD)-10 codes (in total 5,086 males and 4,963 females).

### Outcomes

We subcategorized the main outcome, valvular heart diseases, into the 2 groups, rheumatic valvular heart diseases (ICD-10 codes I05-I09) and nonrheumatic valvular heart diseases (ICD-10 codes I34-I39), and also subdivided them into the different types of valvular heart disease based on anatomical localization. Only first Valvular Heart Disease events were used in the analysis, regardless of type. In total, 218,842 cases were identified, that is, 77,693 inpatients (35.5%), 104,552 outpatients (47.8%), and 36,597 patients from primary health care (16.7%).

### Sociodemographic variables

The population was stratified by *sex*.

*Country of birth* was divided into Swedish-born or foreign-born.

*Age* was used as a continuous variable in the analysis.

*Educational attainment* (for those 0-18 years for parents) was categorized as ≤9 years (partial or complete compulsory schooling), 10 to 12 years (partial or complete secondary schooling), and >12 years (attendance at college and/or university).

*Geographic region of residence* was included to adjust for possible regional differences in hospital admissions and was categorized as: 1) large cities; 2) southern Sweden; and 3) northern Sweden. Large cities were defined as municipalities with a population of >200,000 and comprised the 3 largest cities in Sweden: Stockholm, Gothenburg, and Malmö.

*Neighborhood socioeconomic level* was created using Small Area Market Statistics. The average population in each Small Area Market Statistics neighborhood is approximately 2000 people for Stockholm and 1000 people for the rest of Sweden. A summary index was calculated to characterize neighborhood-level socioeconomic status (SES). The index was categorized into 3 groups: more than one SD below the mean (high SES or low deprivation level), more than 1 SD above the mean (low SES or high deprivation level), and within 1 SD of the mean (middle SES or middle deprivation level).^15^

*Marital status* (for those 0-18 years for parents) was defined as married and not married.

### Comorbidities

We included the following comorbidities as covariates (with ICD-10 codes): hypertension (I10-I19), coronary heart disease (CHD) (I20-I25), cardiomyopathy (I42-I43), atrial fibrillation and flutter (I48), congestive heart disease (congestive heart failure I50, I11.0), stroke (I60-I69), diabetes mellitus (E10-E14), chronic obstructive pulmonary disease (J40-J47), and cancers (C00-C97).

### Statistical analysis

Number of cases of valvular heart diseases was presented by sex and across baseline characteristics. Cox regression analysis was used for estimating HRs with 95% CI of incident valvular heart disease in males compared to females. We also analyzed the different types of rheumatic and nonrheumatic valvular heart diseases. In fully adjusted models, the following covariates were used: age, region of residence in Sweden, educational level, marital status, neighborhood SES, and comorbidities. We also estimated incidence rate ratios. Sensitivity analyses were performed for immigrant status and sector (inpatients, outpatients, primary care) of the health care system as well as analyses that took into account competing risks of death.

## Results

**Figure 1:**
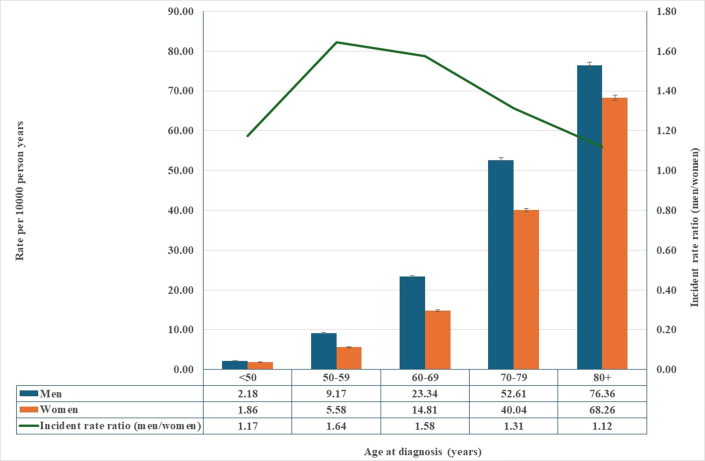
**Incidence of Valvular Heart Diseases by Sex** Age-specific incidence rate (per 100,000 person-years) of valvular heart disease in males and females in Sweden, 1998 to 2018, also including incidence rate ratio (IRR).

**Figure 2:**
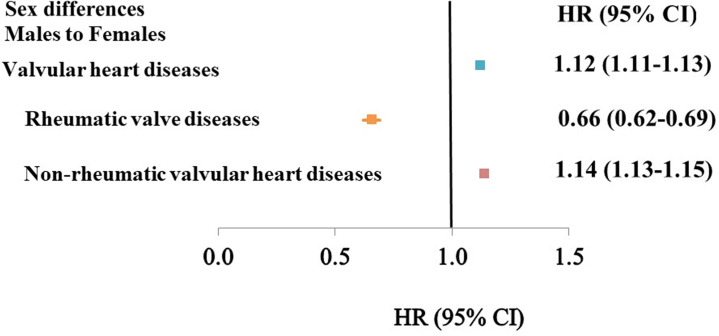
**Incidence of Main Types of Valvular Heart Diseases by Sex in Sweden** Forest chart of association between sex and valvular heart diseases in Sweden, with HRs between men and women (women as referents).

We included 9,984,758 individuals (See Central Illustration), 4,969,472 males and 5,015,286 females (Table 1). In total, 218,842 cases of valvular heart diseases were noted, 111,315 among males and 107,527 among females. The number of cases increased by age (Tables 1 and 2, Figure 1), and the risks for valvular heart diseases were higher for some background factors, such as low and middle educational level, as well as moderate neighborhood deprivation level, but lower among males for high neighborhood deprivation level, and higher for most comorbidities except for stroke (Table 2). There were some differences among rheumatic valvular diseases (Supplemental Table 1) that, in most cases, were similar to the general pattern, and this was also true for nonrheumatic diseases (Supplemental Table 2). In general, valvular diseases were more common in men (Supplemental Figure 2), and this was also true for nonrheumatic diseases (Figure 2), while rheumatic diseases were more common in women. We performed a sex∗age interaction analysis, showing no significant result (*P* = 0.61).

**Central Illustration:**
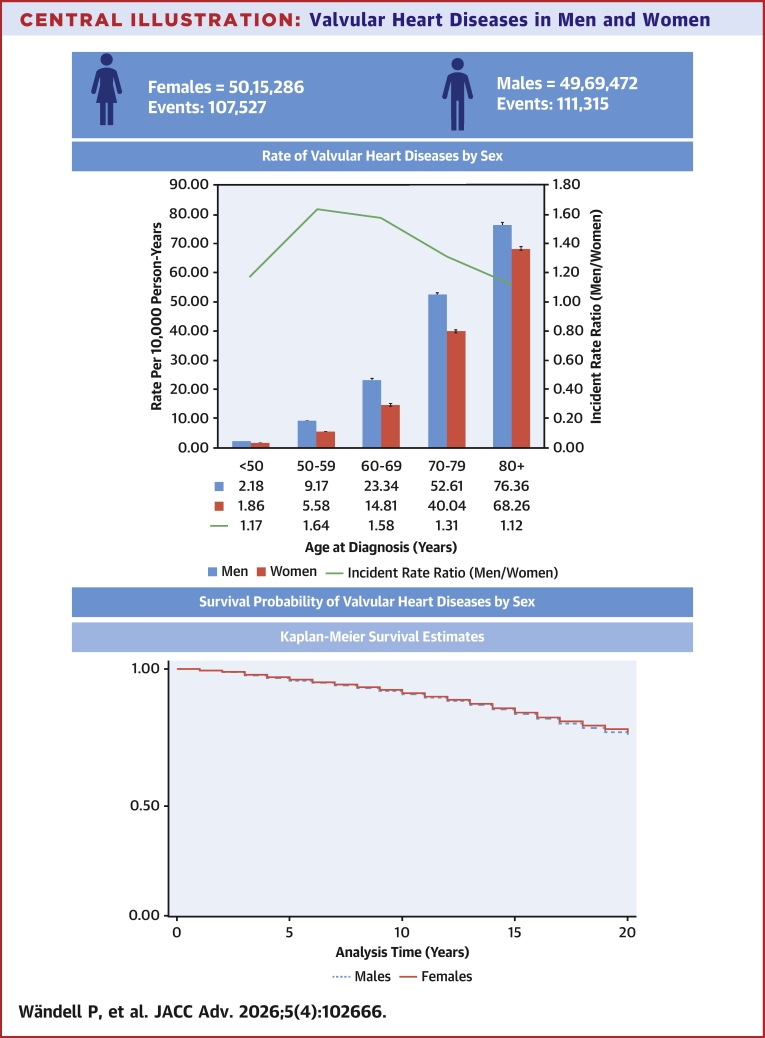
Valvular Heart Diseases in Men and Women

**Table 1:** Study Population and Events of Valvular Heart Disease in Males and Females

	Males		Females	
	Population	Events	Population	Events
Total population	4,969,472	111,315	5,015,286	107,527
Age at diagnosis (y)				
<50		10,925 (9.8)		8,895 (8.3)
50-59		11,390 (10.2)		6,797 (6.3)
60-69		24,105 (21.7)		15,701 (14.6)
70-79		35,967 (32.3)		32,290 (30.0)
80+		28,928 (26.0)		43,844 (40.8)
Educational level				
Low	1,945,884 (39.2)	47,355 (42.5)	1,940,955 (38.7)	52,285 (48.6)
Moderate	1,926,193 (38.8)	41,695 (37.5)	1,876,675 (37.4)	35,858 (33.3)
High	1,097,395 (22.1)	22,265 (20.0)	1,197,656 (23.9)	19,384 (18.0)
Region of residence				
Large cities	2,075,512 (41.8)	52,732 (47.4)	2,155,836 (43.0)	52,750 (49.1)
Southern Sweden	1,451,548 (29.2)	34,650 (31.1)	1,474,406 (29.4)	31,553 (29.3)
Northern Sweden	1,442,412 (29.0)	23,933 (21.5)	1,385,044 (27.6)	23,224 (21.6)
Marital status				
Married/cohabiting	2,921,302 (58.8)	75,202 (67.6)	2,835,123 (56.5)	58,723 (54.6)
Unmarried/widowed/divorced	2,048,170 (41.2)	36,113 (32.4)	2,180,163 (43.5)	48,804 (45.4)
Neighborhood deprivation				
Low	1,001,264 (20.1)	24,966 (22.4)	1,040,214 (20.7)	22,320 (20.8)
Moderate	2,528,753 (50.9)	67,650 (60.8)	2,584,368 (51.5)	65,173 (60.6)
High	1,439,455 (29.0)	18,699 (16.8)	1,390,704 (27.7)	20,034 (18.6)
Immigrant status				
Born in Sweden	3,888,168 (78.2)	99,431 (89.3)	3,950,080 (78.8)	93,736 (87.2)
Born in other countries	1,081,304 (21.8)	11,884 (10.7)	1,065,206 (21.2)	13,791 (12.8)
Comorbidities				
Hypertension	647,013 (13.0)	53,220 (47.8)	682,068 (13.6)	52,702 (49.0)
Coronary heart disease	465,398 (9.4)	44,812 (40.3)	325,678 (6.5)	33,753 (31.4)
Cardiomyopathy	29,021 (0.6)	4,023 (3.6)	16,068 (0.3)	2,429 (2.3)
Atrial fibrillation	337,454 (6.8)	41,584 (37.4)	284,948 (5.7)	35,541 (33.1)
Stroke	305,434 (6.1)	20,040 (18.0)	295,706 (5.9)	18,672 (17.4)
Diabetes	330,442 (6.6)	18,777 (16.9)	262,296 (5.2)	14,763 (13.7)
Chronic obstructive pulmonary disease	260,715 (5.2)	11,609 (10.4)	309,748 (6.2)	13,008 (12.1)
Alcoholism	196,270 (3.9)	4,311 (3.9)	104,665 (2.1)	1385 (1.3)
Cancer	665,231 (13.4)	34,931 (31.4)	668,843 (13.3)	28,226 (26.3)

Values are n (%).

**Table 2:** Risk Factors of Valvular Heart Diseases by Sex

	Males		Females	
	HR^a^ (95% CI)	*P* Value	HR^a^ (95% CI)	*P* Value
Age	**1.05 (1.05–1.05)**	<0.0001	1.05 (1.05–1.05)	<0.0001
Educational level (ref. High)				
Low	**1.07 (1.05–1.08)**	<0.0001	**1.15 (1.13–1.17)**	<0.0001
Moderate	**1.04 (1.02–1.06)**	<0.0001	**1.07 (1.05–1.09)**	<0.0001
Region of residence (ref. Large cities)				
Southern Sweden	**0.89 (0.88–0.91)**	<0.0001	**0.84 (0.82–0.85)**	<0.0001
Northern Sweden	**1.07 (1.05–1.09)**	<0.0001	**1.07 (1.05–1.09)**	<0.0001
Marital status (ref. Married/Cohabiting)	**0.93 (0.92–0.95)**	<0.0001	**0.99 (0.97–1.00)**	0.0248
Neighborhood deprivation (ref. Low)				
Moderate	**1.05 (1.03–1.06)**	<0.0001	**1.07 (1.06–1.09)**	<0.0001
High	**0.95 (0.93–0.97)**	<0.0001	1.00 (0.98–1.02)	0.6972
Immigrant status (ref. Born in Sweden)	1.00 (0.99–1.02)	0.6832	**1.07 (1.05–1.09)**	<0.0001
Comorbidities (ref. Non)				
Hypertension	**1.74 (1.72–1.76)**	<0.0001	**1.70 (1.68–1.72)**	<0.0001
Coronary heart disease	**2.07 (2.04–2.09)**	<0.0001	**2.09 (2.06–2.12)**	<0.0001
Cardiomyopathy	**2.37 (2.29–2.45)**	<0.0001	**2.73 (2.62–2.84)**	<0.0001
Atrial fibrillation	**2.51 (2.48–2.55)**	<0.0001	**2.51 (2.47–2.54)**	<0.0001
Stroke	0.99 (0.98–1.01)	0.4078	1.00 (0.98–1.01)	0.7914
Diabetes	**1.05 (1.03–1.07)**	<0.0001	**1.13 (1.11–1.15)**	<0.0001
Chronic obstructive pulmonary disease	**1.16 (1.14–1.18)**	<0.0001	**1.25 (1.22–1.27)**	<0.0001
Alcoholism	**1.09 (1.05–1.12)**	<0.0001	0.99 (0.94–1.04)	0.6906
Cancer	**1.06 (1.05–1.07)**	<0.0001	**1.07 (1.06–1.09)**	<0.0001

SES = socioeconomic status.

a Fully adjusted (age, region of residence in Sweden, educational level, marital status, neighborhood SES, and comorbidities); **bold** values are statistically significant.

Table 3 shows the HRs for different types of rheumatic and nonrheumatic valvular heart diseases. In total, 9,706 cases of rheumatic valvular diseases were registered (3,808 among males and 5,898 among females), and 209,136 nonrheumatic valvular diseases (107,507 among males and 101,629 among females (Supplemental Table 3). The incidence rates per 100,000 person-years were higher among males for all types of valvular heart diseases (Figure 1), and for nonrheumatic valvular diseases, but lower for rheumatic valvular diseases. The mean age was higher among females (Supplemental Table 3).

**Table 3:** Sex Difference in Incidence of Specific Valvular Heart Diseases in Males Compared to Females (Reference Group)

Diagnosis, ICD-10 Code	Observations in Males	Observations in Females	HR^a^ (95% CI)
Rheumatic valve diseases			
Rheumatic mitral valve diseases I05	1,182	2,376	**0.50 (0.46–0.53)**
Mitral stenosis I05.0	72	213	**0.34 (0.26–0.45)**
Rheumatic mitral regurgitation I05.1	40	56	0.71 (0.47–1.08)
Mitral stenosis with regurgitation I05.2	35	82	**0.40 (0.26–0.60)**
Other, unspecified types I05.8, I05.9	1,035	2,025	**0.51 (0.47–0.55)**
Rheumatic aortic valve diseases I06	1,036	1,082	1.08 (0.99–1.18)
Rheumatic aortic stenosis I06.0	85	95	0.88 (0.65–1.20)
Rheumatic aortic regurgitation I06.1	28	34	0.75 (0.45–1.27)
Rheumatic aortic stenosis with regurgitation I06.2	31	41	0.79 (0.49–1.29)
Other types, I06.8, I06.9	892	912	**1.12 (1.02–1.23)**
Rheumatic tricuspid valve diseases I07	1,118	1,765	**0.61 (0.57–0.66)**
Tricuspid stenosis I07.0	2	5	0.44 (0.08–2.36)
Tricuspid regurgitation I07.1	308	497	**0.60 (0.52–0.69)**
Tricuspid stenosis with regurgitation I07.2	8	11	0.83 (0.31–2.18)
Other types I07.8, I07.9	800	1,252	**0.62 (0.56–0.68)**
Other specified rheumatic heart diseases I08, I09	540	798	**0.73 (0.66–0.82)**
Nonrheumatic valvular heart diseases			
Nonrheumatic mitral valve disorders I34	24,521	22,715	**1.08 (1.06–1.10)**
Mitral (valve) regurgitation I34.0	4,640	5,234	**0.83 (0.79–0.86)**
Mitral (valve) prolapse I34.1	342	270	**1.20 (1.01–1.41)**
Nonrheumatic mitral (valve) stenosis I34.2	76	197	**0.35 (0.26–0.46)**
Other types I34.8, I34.9	19,463	17,014	**1.17 (1.14–1.19)**
Nonrheumatic aortic valve disorders I35	70,691	62,074	**1.25 (1.24–1.27)**
Aortic (valve) stenosis I35.0	9,999	10,638	0.97 (0.95–1.00)
Aortic (valve) regurgitation I35.1	3,480	3,327	1.04 (0.99–1.10)
Aortic (valve) stenosis with regurgitation I35.2	1,465	1,387	**1.09 (1.01–1.18)**
Other types I35.8, I35.9	55,747	45,722	**1.34 (1.32–1.36)**
Nonrheumatic tricuspid valve disorders I36	1,937	2,622	**0.72 (0.68–0.77)**
Nonrheumatic tricuspid (valve) stenosis I36.0	12	19	0.63 (0.30–1.33)
Nonrheumatic tricuspid (valve) regurgitation I36.1	363	619	**0.54 (0.47–0.62)**
Nonrheumatic tricuspid (valve) stenosis with regurgitation I36.2	6	8	0.70 (0.24–2.10)
Other types I36.8, I36.9	1,556	1,976	**0.78 (0.73–0.84)**
Pulmonary valve disorders I37	845	961	**0.82 (0.75–0.90)**
Pulmonary valve stenosis I37.0	51	84	**0.60 (0.42–0.86)**
Pulmonary valve regurgitation I37.1	105	105	0.91 (0.69–1.20)
Pulmonary valve stenosis with regurgitation I37.2	11	10	0.99 (0.42–2.37)
Other types I37.8, I37.9	678	762	**0.83 (0.75–0.92)**
Others I38, I39	11,767	16,250	**0.84 (0.82–0.87)**

IDC = International Classification of Diseases; other abbreviation as in Table 2.

a Fully adjusted (age, region of residence in Sweden, educational level, marital status, neighborhood SES, and comorbidities).

For rheumatic diseases (Table 3), the risk among males for mitral and tricuspid valvular diseases was lower, and also for the group of other valvular diseases, but with no statistically significant sex difference for aortic valvular diseases. For nonrheumatic valvular diseases (Table 3), the risk of mitral and aortic valvular disease was higher in males, while the risk was lower for mitral regurgitation and mitral stenosis, tricuspid valvular disease, pulmonary valvular diseases, and the other subtypes.

The sensitivity analyses of immigrant status (Supplemental Table 4) showed that valvular diseases in general and nonrheumatic valvular diseases were more common in men, and rheumatic valvular diseases were more common in women, in both Swedish- and foreign-born individuals. Regarding diagnoses by health care sector (Supplemental Table 4), we found that valvular diseases in general and nonrheumatic valvular diseases were more common in men, and rheumatic valvular diseases were more common in women, both inpatients and outpatients, while in primary health care, these diseases were more common among women. The sensitivity analysis regarding the number of diagnoses (Supplemental Table 5) showed an increased difference with the number of registered diagnoses (from only 1 to 6 times or more), that is, with increased HR (with 95% CI) for men vs women as regard all valvular diseases (from 0.95 [0.94-0.97] to 1.30 [1.28-1.32]), and nonrheumatic valvular diseases (from 0.97 [0.95-0.99] to 1.35 [1.33-1.37]), and a decreased HR for men vs women for rheumatic valvular diseases (from 0.69 [0.65-0.74] to 0.48 [0.42-0.53]).

A competing risk of death analysis was performed for incidence rates (Supplemental Table 6), with very similar results especially for all valvular heart diseases and nonrheumatic valvular heart diseases, but with some differences for rheumatic valvular heart diseases; fully adjusted models comparing men to women were 0.65 (95% CI: 0.62-0.68), and with adjustment for competing risks 0.91 (95% CI: 0.89-0.93).

## Discussion

The main findings of the present study were that there were sex differences in risk among males compared to females for different types of valvular disorders; for rheumatic valvular disorders, the risk was generally higher among females, except for aortic valvular disorders. For nonrheumatic valvular disorders, the risk was, in general, higher among males, except for some mitral, tricuspid, and pulmonary valvular disorders being more common in females.

The higher risk among females compared to males of being diagnosed with rheumatic mitral and tricuspid valvular disorders, and also pulmonary valvular disorders, has also been found in earlier studies.^5^, ^6^, ^7^, ^8^, ^9^, ^10^, ^11^ The reason why females are more likely to develop valvular diseases on these sites is not known, although some potential mechanisms have been proposed.^6^^,^^16^ An earlier review stated that mitral regurgitation is equally prevalent in males and females, or slightly more prevalent in males, which is in contrast to our findings,^17^ but that rheumatic mitral regurgitation is more common in females, in line with our findings. Differences in extracellular remodeling could be responsible for the sex differences being seen.^5^ Regarding the higher risk in women of tricuspid valvular diseases, it has been proposed that atrial fibrillation could drive annular dilatation to a larger extent among females, thus resulting in secondary tricuspid regurgitation.^5^ The higher risk for pulmonary valvular diseases among females has also been found in earlier Swedish studies, especially for regurgitation.^10^^,^^11^ Interestingly, pulmonary valve stenosis has been reported to be associated with congenital defects in 95% of the cases.^5^ Postmenopausal women are at an increased risk of cardiometabolic diseases in general, and also of valvular heart diseases.^18^ A Japanese study found a higher proportion among females of severe aortic stenosis that increased with age, while in age <65 years, the disease was more common in men.^14^

On the other hand, males seem to be more likely to develop aortic valvular diseases, especially nonrheumatic valvular diseases.^5^^,^^10^^,^^19^ The higher risk of aortic stenosis in men has been suggested to be associated with both a higher risk of bicuspid aorta valve and of endocarditis,^5^ but also with aortic valve calcification earlier in life than in women, and associated with sex hormone patterns.^20^ Furthermore, mineralocorticoid receptor expression also seems to be associated with aortic valve calcification in men, while estrogen treatment blocks valve interstitial cells activation, inflammation, and fibrosis.^21^ However, the risk of calcific degeneration of tricuspid aorta valves increases with age,^5^ and among females, especially postmenopause.^20^ Regarding sex hormones, a U.S. study found that high free estradiol index (estradiol to sex hormone-binding protein) was associated with a higher risk of metabolic syndrome and of higher C-reactive protein in men and women ≥50 years, whereas a higher free androgen index (testosterone to sex hormone-binding protein) was associated with a higher risk of metabolic syndrome and of higher C-reactive protein in women <50 years.^22^ Men are described to have a higher overall risk of developing aortic stenosis, although the disease is predominant among elderly women.^5^^,^^14^ A Danish study showed an increased risk of aortic stenosis and aortic regurgitation but not of mitral stenosis over time, and especially among men and with increasing age.^9^ A Japanese study found a higher proportion of females with severe aortic stenosis, and with increasing rates by age.^14^ Interestingly, the proportion was higher among males compared to females in ages below 65 years, but after that age the female-to-male ratio increased greatly with older age. A Chinese study found sex differences regarding risk factors among elderly individuals with degenerative valvular heart diseases, with higher risks for smoking, chronic obstructive pulmonary disease, CHD, and cardiomyopathies among males, while there were higher risks for hypertension and atrial fibrillation among females.^23^ As males develop CHDs, including myocardial infarction,^24^ atrial fibrillation,^25^^,^^26^ and heart failure,^27^^,^^28^ earlier than females, the higher risk of nonrheumatic valvular heart diseases among males seems expected. A Swedish study found dysglycemia to be associated with a higher risk of aortic stenosis,^29^ and in Sweden, there is a male preponderance for diabetes.^30^ A Danish study found individuals with low SES to be more affected by left heart valvular disease, especially aortic stenosis.^9^

Regarding the sex pattern of valvular heart diseases worldwide, there seem to be both similarities and differences. Globally, rheumatic valvular heart diseases dominate and are most common in low- and middle-income countries.^2^ We found females to have a higher risk for rheumatic valvular diseases, and males for nonrheumatic valvular heart diseases, in line with some earlier findings.^5^ A Danish study found aortic stenosis to be more common than both aortic regurgitation and mitral regurgitation, and the aortic valve disorders to be more common in men, while mitral regurgitation was equally common in men and women.^9^ The earlier cited Chinese study of individuals aged ≥60 years with nonrheumatic heart valvular diseases found aortic diseases to be more common in men, and the opposite for mitral diseases.^23^ However, a recently published study on global nonrheumatic valvular heart diseases among individuals aged 60 to 89 years found a higher risk among females.^31^ In low-income countries, rheumatic heart diseases are more common, especially in children and females,^32^ for example, in Fiji,^12^^,^^33^ India,^34^ and Nicaragua.^13^

The risk was higher in some groups, such as rheumatic valvular diseases among immigrants, which is also described in more detail in a previous article.^10^ In that study, valvular heart diseases in general were more uncommon among foreign-born individuals, while chronic rheumatic valvular heart diseases were more common within that group. Furthermore, we also found lower educational level to be associated with a higher risk of heart valvular diseases, especially among women. The general patterns, with higher rates among men of all valvular diseases and nonrheumatic diseases, and with higher rates among women of rheumatic valvular diseases, were apparent for both in-hospital and out-hospital patients, but not for primary health care patients, where higher rates were found in women for all valvular diseases as well as for rheumatic and nonrheumatic diseases.

For the selected comorbidities, the risk of being diagnosed with valvular heart disease was increased, except for stroke. In addition, there were some minor differences when subdividing the risks into rheumatic and nonrheumatic valvular heart diseases. The higher HRs for cardiomyopathies^35^ and atrial fibrillation^25^ among males have also been described earlier. As both valvular diseases and multimorbidity increase with age,^36^ the association between valvular diseases and other diseases is not surprising.

In the clinical setting, it is important to be aware of the risk of valvular heart diseases not only among men but also in women, especially after menopause. As the risk increases with age, careful heart examinations among the elderly are important. Based on our results, it is difficult to give advice on screening procedures. However, an earlier study showed that an echocardiographic screening could reveal many undiagnosed valvular heart diseases.^37^ As valvular heart diseases increase with age, more efforts could be made for treating the elderly population.

In conclusion, we found different sex patterns for the various kinds of heart valvular diseases. There was a male predominance overall for valvular heart diseases, especially for nonrheumatic valvular diseases, but with a higher risk among females compared to males for certain specific valvular heart diseases, including rheumatic mitral and tricuspid valvular diseases, nonrheumatic tricuspid valvular diseases, and pulmonary valvular diseases. The underlying factors behind the sex differences are, in many cases, unknown, even if some background risk factors, including socioeconomic factors, could be of importance and need further investigation.

### Study Limitations

This study has some limitations. We used the NPR with diagnoses from electronic patient registers and with no possibility to check whether diagnostic criteria had been used, although many diagnoses have been validated.^38^^,^^39^ Additionally, we did not have access to data on the severity of the different valvular heart diseases. Furthermore, the number of separate diagnoses in the categories of “other types” is high for most heart valves, although the risk estimates were rather similar in the different subtypes of valvular heart diseases.

The study also has many strengths. For example, we used national Swedish data, and the Swedish registers have been shown to have good quality.^38^^,^^39^ The Swedish personal identity numbers (replaced by pseudonymized serial numbers) allow linkage between different registers,^40^ thus enabling adjustments for many potentially confounding factors.

## Funding support and author disclosures

This work was supported by grants from the Swedish Heart Lung foundation (awarded to Dr Sundquist, 20241289), the 10.13039/501100004359Swedish Research Council (to Dr Sundquist, 2022-00812), and ALF funding from 10.13039/501100009780Region Skåne. The authors have reported that they have no relationships relevant to the contents of this paper to disclose.
