# Supplementary material for: Valvular Heart Diseases in Swedish Males and Females: A National Cohort Study
Source: JACC Adv. 2026 Mar 19;5(4):102666. doi: 10.1016/j.jacadv.2026.102666 (PMC13019594; doi:10.1016/j.jacadv.2026.102666)

| **Supplementary Table S1. Risk factors for rheumatic valvular heart disease by gender** | | | |  |  |  |  |  |  |  |
| --- | --- | --- | --- | --- | --- | --- | --- | --- | --- | --- |
|  | Males | | | |  | Females | | | |  |
|  | HR* | 95% CI | | P-value |  | HR* | 95% CI | | P-value |  |
| **Age** | **1.04** | **1.04** | **1.04** | <.0001 |  | **1.03** | **1.03** | **1.03** | <.0001 |  |
| **Educational level (ref. High)** |  |  |  |  |  |  |  |  |  |  |
| Low | **1.15** | **1.05** | **1.26** | 0.0025 |  | **1.27** | **1.18** | **1.37** | <.0001 |  |
| Moderate | 1.08 | 0.98 | 1.18 | 0.1195 |  | 1.03 | 0.96 | 1.11 | 0.4313 |  |
| **Region of residence (ref. Large cities)** |  |  |  |  |  |  |  |  |  |  |
| Southern Sweden | 1.04 | 0.97 | 1.13 | 0.2557 |  | 1.01 | 0.95 | 1.07 | 0.7276 |  |
| Northern Sweden | **1.16** | **1.06** | **1.26** | 0.0009 |  | **1.10** | **1.03** | **1.18** | 0.0047 |  |
| **Marital status (ref. Married/Cohabiting)** | 1.00 | 0.93 | 1.07 | 0.9113 |  | **0.92** | **0.88** | **0.97** | 0.0026 |  |
| **Neighborhood deprivation (ref. Low)** |  |  |  |  |  |  |  |  |  |  |
| Moderate | 0.97 | 0.89 | 1.06 | 0.5053 |  | 1.03 | 0.96 | 1.10 | 0.3963 |  |
| High | **1.13** | **1.02** | **1.25** | 0.0224 |  | **1.25** | **1.15** | **1.35** | <.0001 |  |
| **Immigrant status (ref. Born in Sweden)** | **1.63** | **1.49** | **1.78** | <.0001 |  | **1.80** | **1.69** | **1.92** | <.0001 |  |
| **Comorbidities (ref. Non)** |  |  |  |  |  |  |  |  |  |  |
| Hypertension | **1.23** | **1.14** | **1.32** | <.0001 |  | **1.11** | **1.04** | **1.17** | 0.0006 |  |
| Coronary heart disease | **1.64** | **1.52** | **1.76** | <.0001 |  | **1.64** | **1.54** | **1.74** | <.0001 |  |
| Cardiomyopathy | **2.83** | **2.43** | **3.29** | <.0001 |  | **2.63** | **2.22** | **3.11** | <.0001 |  |
| Atrial fibrillation | **4.51** | **4.19** | **4.85** | <.0001 |  | **6.10** | **5.74** | **6.48** | <.0001 |  |
| Stroke | 0.93 | 0.85 | 1.02 | 0.1092 |  | 1.06 | 0.99 | 1.14 | 0.0920 |  |
| Diabetes | 1.04 | 0.95 | 1.13 | 0.4474 |  | **1.16** | **1.08** | **1.26** | <.0001 |  |
| Chronic obstructive pulmonary disease | **1.46** | **1.32** | **1.60** | <.0001 |  | **1.49** | **1.39** | **1.61** | <.0001 |  |
| Alcoholism | 1.14 | 0.97 | 1.33 | 0.1141 |  | **1.26** | **1.04** | **1.52** | 0.0211 |  |
| Cancer | 1.03 | 0.96 | 1.11 | 0.432 |  | 1.03 | 0.97 | 1.10 | 0.3133 |  |
| *: Fully adjusted; bold values statistically significant |  |  |  |  |  |  |  |  |  |  |

| **Supplementary Table S2. Risk factors of non-rheumatic valvular heart disease by sex** | | | |  |  |  |  |  |  |  |
| --- | --- | --- | --- | --- | --- | --- | --- | --- | --- | --- |
|  | Men | | | |  | Females | | | |  |
|  | HR* | 95% CI | | P-value |  | HR* | 95% CI | | P-value |  |
| **Age** | **1.05** | **1.05** | **1.05** | <.0001 |  | **1.05** | **1.05** | **1.05** | <.0001 |  |
| **Educational level (ref. High)** |  |  |  |  |  |  |  |  |  |  |
| Low | **1.06** | **1.05** | **1.08** | <.0001 |  | **1.14** | **1.12** | **1.16** | <.0001 |  |
| Moderate | **1.04** | **1.02** | **1.06** | <.0001 |  | **1.07** | **1.05** | **1.09** | <.0001 |  |
| **Region of residence (ref. Large cities)** |  |  |  |  |  |  |  |  |  |  |
| Southern Sweden | **0.89** | **0.88** | **0.90** | <.0001 |  | **0.83** | **0.81** | **0.84** | <.0001 |  |
| Northern Sweden | **1.07** | **1.05** | **1.08** | <.0001 |  | **1.06** | **1.05** | **1.08** | <.0001 |  |
| **Marital status (ref. Married/Cohabiting)** | **0.93** | **0.92** | **0.94** | <.0001 |  | 0.99 | 0.98 | 1.00 | 0.1517 |  |
| **Neighborhood deprivation (ref. Low)** |  |  |  |  |  |  |  |  |  |  |
| Moderate | **1.05** | **1.04** | **1.07** | <.0001 |  | **1.08** | **1.06** | **1.09** | <.0001 |  |
| High | **0.94** | **0.92** | **0.96** | <.0001 |  | 0.99 | 0.97 | 1.01 | 0.2385 |  |
| **Immigrant status (ref. Born in Sweden)** | 0.98 | 0.96 | 1.00 | 0.0809 |  | **1.02** | **1.00** | **1.04** | 0.0178 |  |
| **Comorbidities (ref. Non)** |  |  |  |  |  |  |  |  |  |  |
| Hypertension | **1.76** | **1.74** | **1.78** | <.0001 |  | **1.74** | **1.71** | **1.76** | <.0001 |  |
| Coronary heart disease | **2.08** | **2.05** | **2.11** | <.0001 |  | **2.12** | **2.09** | **2.15** | <.0001 |  |
| Cardiomyopathy | **2.35** | **2.27** | **2.43** | <.0001 |  | **2.74** | **2.63** | **2.86** | <.0001 |  |
| Atrial fibrillation | **2.46** | **2.43** | **2.50** | <.0001 |  | **2.39** | **2.35** | **2.42** | <.0001 |  |
| Stroke | 1.00 | 0.98 | 1.01 | 0.5683 |  | 0.99 | 0.98 | 1.01 | 0.4667 |  |
| Diabetes | **1.05** | **1.03** | **1.07** | <.0001 |  | **1.13** | **1.11** | **1.15** | <.0001 |  |
| Chronic obstructive pulmonary disease | **1.15** | **1.13** | **1.17** | <.0001 |  | **1.23** | **1.21** | **1.26** | <.0001 |  |
| Alcoholism | **1.09** | **1.05** | **1.12** | <.0001 |  | 0.97 | 0.92 | 1.03 | 0.3277 |  |
| Cancer | **1.06** | **1.05** | **1.08** | <.0001 |  | **1.08** | **1.06** | **1.09** | <.0001 |  |
| *: Fully adjusted.; bold values are statistically significant |  |  |  |  |  |  |  |  |  |  |

| **Supplementary Table S3. Study population and mean age of diagnosis in males and females** | | | |  |
| --- | --- | --- | --- | --- |
|  | Males |  | Females | |
| Study population | 4969472 |  | 5015286 | |
| Number of events | 111315 |  | 107527 | |
| Rheumatic valve diseases | 3808 |  | 5898 | |
| Non-rheumatic valve diseases | 107507 |  | 101629 | |
| Mean age at diagnosis (years) (± SD) | 69.4 (15.0) |  | 73.4 (15.3) | |
| Rheumatic valve diseases | 66.4 (18.5) |  | 69.5 (18.1) | |
| Non-rheumatic valve diseases | 69.5 (14.9) |  | 73.6 (15.1) | |
| Incidence rate per 100000 person years (95% CI) | 68.6 (67.5-69.8) |  | 51.3 (50.6-51.9) | |
| Rheumatic valve diseases | 2.6 (2.5-2.7) |  | 3.3 (3.2-3.4) | |
| Non-rheumatic valve diseases | 66.0 (65.6-66.4) |  | 48.0 (47.7-48.3) | |

| **Supplementary Table S4. Gender difference in incidence of the different valvular heart diseases in Swedish-born and foreign-born, respectively, and in the different sectors of the health-care system, in men compare to women (reference group) expressed as hazard ratios (HR) with 95% confidence intervals (95% CI)** | | | | | | |
| --- | --- | --- | --- | --- | --- | --- |
|  | Obs in men | Obs in women |  | HR* | 95% CI | |
| **Immigrant status:** |  |  |  |  |  |  |
| Swedish-born |  |  |  |  |  |  |
| **Valvular heart diseases** | 99431 | 93736 |  | 1.13 | 1.12 | 1.14 |
| **Rheumatic valve diseases:** | 3152 | 4631 |  | 0.70 | 0.67 | 0.74 |
| **Non-rheumatic valvular heart diseases** | 96279 | 89105 |  | 1.16 | 1.14 | 1.17 |
| Foreign-born |  |  |  |  |  |  |
| **Valvular heart diseases** | 11884 | 13791 |  | 1.03 | 1.00 | 1.05 |
| **Rheumatic valve diseases:** | 656 | 1267 |  | 0.54 | 0.49 | 0.59 |
| **Non-rheumatic valvular heart diseases** | 11228 | 12524 |  | 1.08 | 1.05 | 1.11 |
|  |  |  |  |  |  |  |
| **Sector of health-care system:** |  |  |  |  |  |  |
| In-patient diagnosis |  |  |  |  |  |  |
| **Valvular heart diseases** | 41048 | 36645 |  | 1.23 | 1.21 | 1.25 |
| **Rheumatic valve diseases:** | 1753 | 2588 |  | 0.73 | 0.69 | 0.78 |
| **Non-rheumatic valvular heart diseases** | 39295 | 34057 |  | 1.27 | 1.25 | 1.29 |
| Out-patient diagnosis |  |  |  |  |  |  |
| **Valvular heart diseases** | 55308 | 49244 |  | 1.17 | 1.16 | 1.19 |
| **Rheumatic valve diseases:** | 1619 | 2584 |  | 0.60 | 0.57 | 0.64 |
| **Non-rheumatic valvular heart diseases** | 53689 | 46660 |  | 1.21 | 1.19 | 1.22 |
| Primary health care diagnosis |  |  |  |  |  |  |
| **Valvular heart diseases** | 14959 | 21638 |  | 0.80 | 0.78 | 0.82 |
| **Rheumatic valve diseases:** | 436 | 726 |  | 0.65 | 0.58 | 0.73 |
| **Non-rheumatic valvular heart diseases** | 14523 | 20912 |  | 0.80 | 0.79 | 0.82 |
| *: Fully adjusted. |  |  |  |  |  |  |

| **Supplementary Table S5. Number of diagnoses of valvular heart disease during the study period in men and women, and risks of gender difference** | | | | | | | | |  |
| --- | --- | --- | --- | --- | --- | --- | --- | --- | --- |
| Number of diagnosis | Men | |  | Women | |  | Risk for men (ref. Women) | | |
|  | No. | % |  | No. | % |  | HR* | 95% CI | |
| **Valvular heart diseases** |  |  |  |  |  |  |  |  |  |
| 1 (only once of valvular heart disease) | 26687 | 24.0 |  | 30631 | 28.5 |  | 0.95 | 0.94 | 0.97 |
| 2 | 19144 | 17.2 |  | 20779 | 19.3 |  | 1.04 | 1.02 | 1.06 |
| 3 | 9225 | 8.3 |  | 9579 | 8.9 |  | 1.07 | 1.03 | 1.10 |
| 4 | 8581 | 7.7 |  | 8181 | 7.6 |  | 1.16 | 1.12 | 1.19 |
| 5 | 5560 | 5.0 |  | 5113 | 4.8 |  | 1.19 | 1.14 | 1.23 |
| 6+ | 42118 | 37.8 |  | 33244 | 30.9 |  | 1.30 | 1.28 | 1.32 |
| **Rheumatic valve disease** |  |  |  |  |  |  |  |  |  |
| 1 (only once of rheumatic valve disease) | 1506 | 39.5 |  | 2291 | 38.8 |  | 0.69 | 0.65 | 0.74 |
| 2 | 1208 | 31.7 |  | 1697 | 28.8 |  | 0.76 | 0.70 | 0.82 |
| 3 | 263 | 6.9 |  | 408 | 6.9 |  | 0.65 | 0.55 | 0.76 |
| 4 | 255 | 6.7 |  | 410 | 7.0 |  | 0.62 | 0.53 | 0.73 |
| 5 | 93 | 2.4 |  | 162 | 2.7 |  | 0.57 | 0.44 | 0.74 |
| 6+ | 483 | 12.7 |  | 930 | 15.8 |  | 0.48 | 0.42 | 0.53 |
| **Non-rheumatic valvular heart diseases** |  |  |  |  |  |  |  |  |  |
| 1 (only once of non-rheumatic valvular heart disease) | 25671 | 23.9 |  | 29026 | 28.6 |  | 0.97 | 0.95 | 0.99 |
| 2 | 18384 | 17.1 |  | 19545 | 19.2 |  | 1.06 | 1.04 | 1.09 |
| 3 | 8909 | 8.3 |  | 9106 | 9.0 |  | 1.08 | 1.05 | 1.12 |
| 4 | 8354 | 7.8 |  | 7836 | 7.7 |  | 1.18 | 1.14 | 1.21 |
| 5 | 5420 | 5.0 |  | 4899 | 4.8 |  | 1.21 | 1.16 | 1.26 |
| 6+ | 40769 | 37.9 |  | 31217 | 30.7 |  | 1.35 | 1.33 | 1.37 |
| *: Fully adjusted. |  |  |  |  |  |  |  |  |  |

| **Supplementary Table S6. Gender difference in incidence of specific valvular heart diseases in men compared 95% CI)** | | | | | | | |
| --- | --- | --- | --- | --- | --- | --- | --- |
|  | Fully adjusted | | |  | Adjusted for competing risk | | |
| Diagnosis | HR* | 95% CI | |  | HR* | 95% CI | |
| **All Valvular heart diseases** | 1.115 | 1.106 | 1.125 |  | 1.117 | 1.108 | 1.127 |
| **Rheumatic valvular heart diseases** | 0.651 | 0.624 | 0.678 |  | 0.909 | 0.887 | 0.931 |
| **Non-rheumatic valvular heart diseases** | 1.144 | 1.133 | 1.154 |  | 1.143 | 1.133 | 1.153 |
| *: Fully adjusted. |  |  |  |  |  |  |  |

**Supplementary Figure S1.** Age-specific incidence rate (per 100 000 person years) of valvular heart disease in males and females


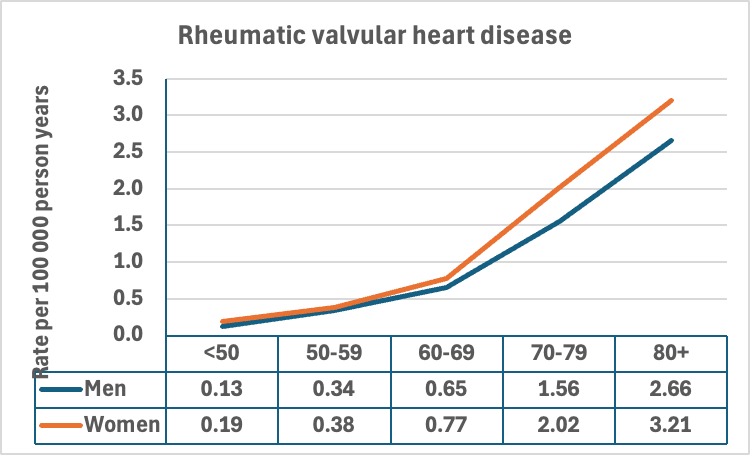

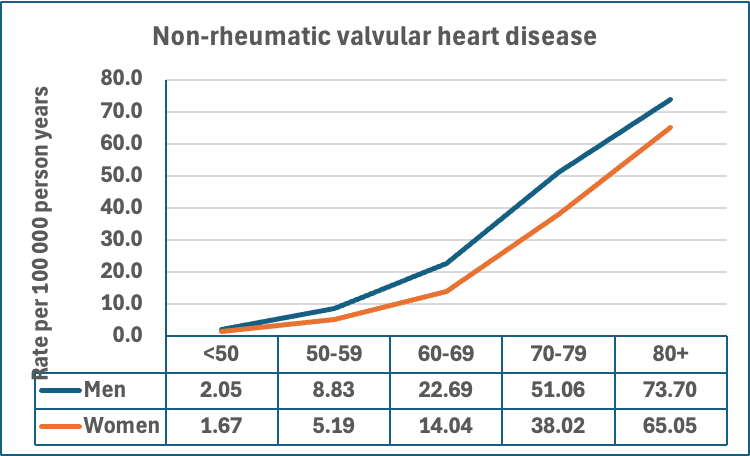


**Supplementary Figure S2. Kaplan-Meier curves showing the cumulative incidence of any valvular heart disease diagnosis in males and females during the study period (1998-2018).**
Log-rank p-value < 0.0001.


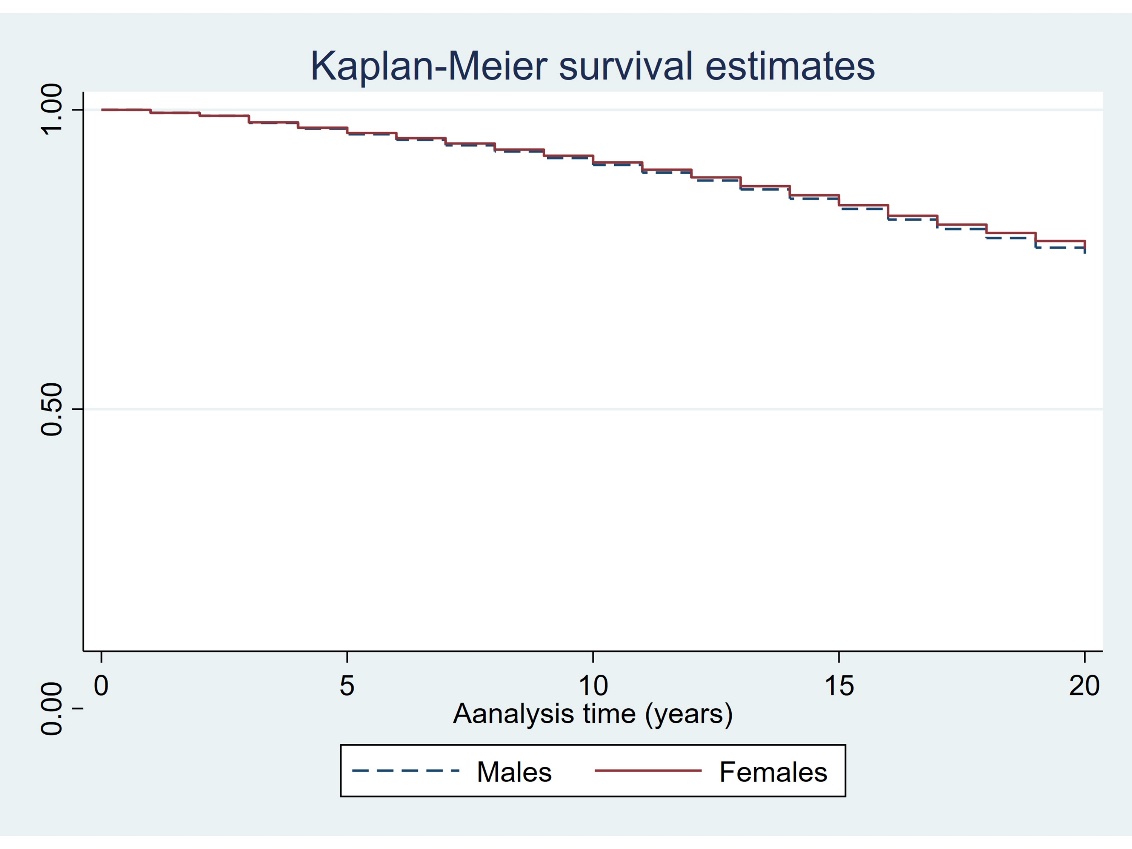

Supplement: Supplemental Tables 1-6 and Supplemental Figures 1 and 2 [file mmc1.docx]
